# The 10th anniversary of the Paris agreement on climate: unmet goals and unanswered questions

**DOI:** 10.3389/fpubh.2025.1657860

**Published:** 2025-10-29

**Authors:** Paolo Vineis, Raimondo Orsini, Giorgio Vacchiano

**Affiliations:** ^1^Imperial College London, London, United Kingdom; ^2^Accademia dei Lincei, Roma, Italy; ^3^Fondazione per lo Sviluppo Sostenibile, Roma, Italy; ^4^Università Statale, Milano, Italy

**Keywords:** COP, mitigation solutions, nationally determined contributions, technology, co-benefits, low-income countries, health

## Abstract

The contribution of interventions for mitigation of climate change in different sectors has been evaluated by IPCC in their 6th report (AR6) and by the International Energy Agency (IEA), among others. However, these scientific evaluations have not been translated into a systematic, overall appraisal, that combines the few quantified targets, such as greenhouse gas emissions and temperature trajectories, with broader but less quantified dimensions such as feasibility, equity, justice, co-benefits (including health), and acceptability. These aspects remain fragmented or insufficiently assessed at the global scale, as a large number of questions are still open on the suitability of different mitigation measures. Progress has been made in certain sectors, such as the phase-out of coal in many countries; the first substantial consideration of health at COP27 and the growing attention to its impacts; the renewed focus on Net Zero commitments; the launch of the European Union's “Fit for 55” package, toward which the EU is nearly on track as of 2025; and the recognition of the need to connect climate action with local benefits, including for health. But such progress is still insufficient. The purpose of this perspective is to identify gaps in a coordinated international action, and in particular the open questions concerning priorities and feasibility of different mitigation strategies, with special emphasis on health.

## The overall goals of the Paris agreement

Under the Paris Agreement (December 2015), it was established that countries develop and communicate their plans to meet greenhouse gas (GHG) emission reduction targets through their Nationally Determined Contributions (NDCs) (1). To date, the NDCs from all countries combined will not be sufficient to meet the least ambitious Paris Agreement target of achieving a 66% chance of staying below 2 °C by the end of this century (2, 3). In fact, in 2024 the threshold of 1.5 °C has been already overcome, though temporarily. While this does not yet represent a permanent overshoot, it signals the increasing likelihood of breaching the limit consistently in the near future (4).

We are currently facing two gaps: one is the “implementation gap” between actual reductions and what was pledged by each country in their NDCs; the second is that the level of ambition of the NDCs themselves is far from being sufficient to meet the Paris targets (“ambition gap”) (2).

Before the Ukraine war-related energy crisis in 2022, the energy production sector was responsible for approximately 34% of the total net anthropogenic GHG emissions worldwide [20 gigatonnes of CO_2_-equivalent per year (GtCO_2_-eq y^−1^)], industry accounted for 23% (14 GtCO_2_-eq y^−1^), agriculture, forestry and other land use (AFOLU) for 22% (13 GtCO_2_-eq y-1), transport for 15% (8.7 GtCO_2_-eq y^−1^) and the remaining 6% (3.3 GtCO_2_-eq y^−1^) was attributable to buildings (5). Achieving Net Zero emissions by mid-century is the overarching goal, yet the pathways to reach it vary significantly depending on the sectors involved. Feasibility of mitigation differs widely across sectors, being more feasible in the case of transition to renewable electricity production or electric mobility than in other sectors (6). Sectors such as cement and steel manufacturing, aviation, and agriculture and land use (AFOLU) present particular challenges (7). These include the technical difficulty of decarbonizing industrial processes, the lack of scalable alternatives for long-distance transport, the inefficiency of alternative fuels for aviation, and the dependency of AFOLU transitions on demographic trends, agricultural productivity, food systems, land competition, and reducing waste (8). For these reasons, carbon dioxide removal (CDR) strategies including afforestation, bioenergy with carbon capture and storage (BECCS), enhanced soil carbon sink, or direct air capture are an essential components of Net Zero pathways in most scenarios developed by the IPCC and the IEA (9).

However, while CDR is required to counterbalance hard-to-abate residual emissions, it cannot replace deep emission reductions. The IPCC AR6 concludes that CDR is “unavoidable” for achieving net zero, though only as a complement to rapid emission cuts. In 1.5 °C-consistent pathways, the median cumulative CO_2_ captured and stored by 2100 is ~665 Gt. Out of seven Illustrative Mitigation Pathways by IPCC AR6, only one excludes CCS, but it demands nearly halving global energy demand by mid-century. Even the renewables-intensive pathway still depends on more than 3 GtCO_2_ per year of capture by 2050 (10). The latest global assessment estimates that by 2050, realistic deployment of carbon dioxide removal could offset only about 7–9 GtCO_2_eq of current global emissions, highlighting its limited role compared with the scale of reductions needed (10). A conservative framing is that ~80% of the mitigation effort must come from emission cuts, while removals can contribute at most ~20%, with high uncertainty and a risk of overestimation. On the other hand, high levels of CDR may enable greater fossil fuel consumption (lock-in risk), especially in the short to medium term, before declining renewable energy costs allow CDR to be powered by clean energy. Additionally, the impacts on land use may be very significant: in an extreme “high mitigation + very high CDR” scenario, agricultural land area decreases by up to ~85.9% between 2050 and 2100 (11). These results confirm that CDR should be used as a complement to, not a substitute for, emissions reductions.

## Gap between setting goals and current trends

Current data and trends on emissions cast doubts about the capacity of global society to meet the climate deadlines that all countries subscribed 10 years ago. The recent document called “2030 Climate Solutions—Implementation Roadmap” published by UNFCCC after COP28 (12) has set very ambitious goals, which include:

“*Zero emission vehicles make up 100% of total global passenger vehicles & vans sales by 2030 in key markets”* (page 25)

“*100% plastic packaging is reusable, recyclable, or compostable by 2025”* (page 32)

“*By 2030: climate-resilient, sustainable agriculture is the most attractive and widely adopted option for farmers everywhere and 2 billion hectares of land are sustainably managed. 50% of food globally is produced through sustainable agriculture practices (including agroecological and regenerative approaches), without expansion of the agricultural frontier into pristine ecosystems, (…). By 2030, end hunger and climate-induced malnutrition in all its forms.”* (page 48)

However, the overall ambitions of the report (12) clash with the stark reality described by the report itself:

- Clean power: “*Progress is slower than what is needed, and is uneven, with most of the investment being currently focused on developed countries and China (…). The power sector is not yet on track for net zero by mid-century, although the deployment and manufacture of key technologies have accelerated considerably in recent years. If current rates of growth in wind and solar generation continue, they are set to achieve more than half of what is required by 2030 to get on track for a net zero scenario.”* (page 12)
- Electrification*: “showing an acceleration but at a pace not fast enough to achieve 30% by 2030,”* (page 13) which was set by COP27.
- Oil and gas: “*Investment in Fossil Fuels increased in 2022 and oil production in 2022 was 2.7% below 2019 levels. Meanwhile, fossil-fuel subsidies surged to a record USD 7 trillion in 2022 as governments supported consumers and businesses during the global spike in energy prices. Both explicit and implicit subsidies are currently well above 2019 levels (explicit: USD 1.3 Trillion, 2.6 times the 2019 value).”* (page 20) Such subsidies distort energy markets by making fossil fuels appear cheaper than they are, slowing down the energy transition and diverting resources from renewables. They also increase the risk of stranded assets, i.e, investments in fossil fuel infrastructure that may lose value as global climate policies tighten but would be difficult to divert due to the need for long-term break-even.
- Plastics: “*Plastic waste is projected to triple by 2060, with 50% of all plastic waste still being landfilled, and only 17% being recycled.”* (page 39) Plastics are a major driver of fossil fuel demand, as over 99% of plastics are derived from petrochemicals. Their production and incineration generate significant GHG emissions, while their persistence in the economy reinforces dependency on fossil-based value chains.
- Pharma/Med Tech: “*The sector, while actively engaging in various emission reduction initiatives, has witnessed a rise in its global carbon emissions share from 3.9% in 2021 to 5% in 2022, indicating the urgency for more robust actions.”* (page 44)
- Halting deforestation: “*The Forest Declaration Assessment of 2023 calculates that the world lost 6.6 million hectares of forest, an area larger than Sri Lanka, and deforestation rates increased by 4% in 2022.”* (page 51) Subsequent reports showed a decrease of deforestation, both on a yearly and on a decadal basis, however we are still not on track to meet the Zero Deforestation Goal before 2030 set by COP26.
- Agriculture: “*As a proxy, the organic share of total agricultural land was at 1.5% in 2019, as reported by FIBL, indicating that adoption of sustainable agriculture practices is still very low.”* (page 52)
- Wetlands: “*Wetlands are being lost at alarming rates with 35% loss globally since 1970, wetlands are our most threatened ecosystem, disappearing three times faster than forests.”* (page 66)
- Built environment*: “Since 2015, building sector emissions have increased by 1% annually, driven by global floor area growth, outweighing efficiency gains. Specific indicators of energy efficiency and carbon intensity are not on track.”* (page 74)
- And finally, finance:

“*the provision of international public finance for adaptation declined by 15% (…) Nearly all adaptation finance tracked was funded by public actors (98%) with development finance climate portfolios increasingly prioritizing adaptation.”* (page 86)

“*Despite the urgent need to decarbonise, most of the conversation to mobilize public climate finance still rests on ‘billions.' (..) This capital reallocation is underway but is not occurring at the pace or scale needed.”* (page 90)

“*The ten countries most affected by climate change between 2000 and 2019 received just USD 23 billion; less than 2% of total climate finance.”* (page 92)

Implementing Net Zero strategies at the global level requires a massive scale-up of climate finance, with estimates ranging from $4 to $6 trillion annually by 2030 to support mitigation, adaptation, and a just transition.

The gaps in climate finance are particularly worrying. Under the Paris Agreement, developed countries reaffirmed the commitment first made at COP15 in Copenhagen, 2009, to mobilize at least USD 100 billion annually by 2020 to support developing countries in mitigation and adaptation, including provisions for technology transfer, capacity building, and the creation of a common transparency framework for reporting. This commitment has been repeatedly postponed and, even if partially met in 2022–23 (up to USD 83 billion, mostly delivered as non-concessional loans, and only in a minor part as grants), its volume and quality fell short of both the scale required and the principle of equity. Individual contribution to the Climate Fund, through either bilateral flows or multilateral development banks, are not disclosed by official data sources for climate finance (i.e., OECD). However, recent analyses have reconstructed finance flows, and compared them against a “fair share” quota proportional to the historical cumulative emissions of Annex II countries, i.e., “industrialized” nations legally obliged to provide climate financing under the terms of the UN climate convention. Data shows, for example, that in 2020 the US contributed only 19% of its fair share of the $100bn target, falling $32bn short, while Canada and Australia also contributed less than 40% of their fair share. In contrast, Germany, France and Japan contributed more than their proportional share, though often through loans rather than grants (13).

In the following COPs, additional mechanisms were introduced: COP26 (Glasgow) launched the Glasgow Dialogue on Loss and Damage; COP27 (Sharm el-Sheik) agreed to establish a dedicated Loss and Damage Fund; and COP29 adopted a New Collective Quantified Goal (NCQG), tripling public climate finance for developing countries to USD 300 billion per year by 2035, rising to USD 1.3 trillion annually when including domestic and private-sector contributions. This goal also included the operationalization of the Loss and Damage Fund and replenishment of the Green Climate Fund and the Adaptation Fund. However, many low- and middle-income countries argue that these figures remain insufficient compared to needs and to the historical responsibility of high-income nations. Independent assessments confirm this view. The Climate Action Tracker (CAT) rates international climate finance from most donor countries as “Insufficient” or “Highly insufficient,” with only a handful showing progress. CAT's fair share methodology shows that, despite partial increases in pledges, the aggregate support remains far below what would be consistent with limiting warming to 1.5 °C (14).

Transparency and reporting are also uneven. The Paris Agreement established an Enhanced Transparency Framework, requiring all Parties to submit Biennial Transparency Reports (BTRs) on progress toward their Nationally Determined Contributions. While most developed countries have submitted at least one BTR, coverage and quality vary significantly, especially among low- and middle-income countries, where capacity constraints persist. The first Global Stocktake in 2023 highlighted these gaps, showing that data are often incomplete, incomparable across countries, or delayed, undermining accountability and the collective assessment of progress.

These are striking examples of the discrepancy, if not huge gap, between the ambitious targets that have been set called “solutions” in the document (12) and the reality of progress. This mismatch is not surprising, but it is nonetheless alarming. Cross-national evidence shows that while climate-policy ambition (measured via a new output-based index across 35 major emitters) rose substantially after Paris, performance indicators lagged—revealing a persistent ambition–implementation gap, especially at the domestic policy level (15). At the same time, focusing too narrowly on whether we meet specific targets by 2030 or 2050 may obscure the deeper imperative: to act with determination regardless of the timeline, because every tenth of a degree in avoided warming makes a tangible difference for ecosystems, economies, and human lives.

In this light, it becomes even more urgent to identify not only the gaps, but the concrete levers that can help close them. At least five components are missing or weak: (a) a hierarchy on the relative importance of different interventions, both at global and state level (consistent with national circumstances); (b) the identification of high priority technological gaps; (c) a political roadmap to achieve the goals and establish clear responsibilities; (d) a transparent economic evaluation of the needed resources, with concrete commitments to finance global climate action and monitor its delivery; (e) inclusion of social and health co-benefits evaluation in the overall strategy.

## Economic and political barriers

Since the Stern Review (16) it has been clear that the costs of not acting to tackle climate change are far higher than the costs of mitigation. More recent work confirms this point: the Global Commission on the Economy and Climate estimated that shifting to a low-carbon growth path could generate a $26 trillion economic windfall and over 65 million jobs by 2030 compared to business-as-usual (17). Decarbonising even “hard-to-abate” sectors is technically feasible with existing technologies, at a cost of less than 0.5% of global GDP by 2050 (18).

Conversely, the macroeconomic damage of climate inaction is profound. Damages could reach 10%−20% of global GDP by 2050, with a social cost of carbon exceeding $1,000 per ton (19, 20). Limiting warming to 1.5° C would cut these losses by two-thirds (21). A recent synthesis by the Boston Consulting Group and theUniversity of Cambridge suggests cumulative economic output could shrink by 15%−34% under a +3° C world, compared with 1%−2% of GDP investment needed for mitigation and adaptation (22), i.e., less than current and much less than planned global military expenditure.

Despite the overwhelming economic case for climate action, progress is slow due to structural barriers. These include: (i) limited understanding among leaders of the economic damages of climate change, which undermine growth, health, and security; (ii) the mismatch between near-term costs of action and the long-term benefits, most evident after 2050; (iii) uneven distribution of costs and benefits between countries, with no consensus on equitable emission reductions; (iv) domestic winners and losers that necessitate a just transition; and (v) persistent uncertainty and underestimation of damages, especially regarding extremes and tipping points (23). Overcoming these barriers requires reframing climate policy debates around the net costs of inaction, strengthening national climate policies, reinvigorating international cooperation, and ensuring equitable development pathways. Moreover, recent work in political science (24) on “decarbonization states,” i.e., governments that promise to cut emissions quickly but fail to deliver the results, attribute such failure to a perceived conflict with the state's basic responsibilities: keeping the economy growing, making sure citizens feel treated fairly, and protecting national security. So far, most policies have focused on “shift” strategies: switching electricity production from fossil fuels to renewables, replacing petrol cars with electric ones, or promoting plant-based diets instead of meat-heavy ones. These measures are important, but they still assume that the overall level of energy use and resource consumption will stay high. This disrupts existing industries and raises economic costs during the transition, creating resistance from businesses and workers. It is necessary then to add “avoid” measures alongside “shift” ones, reducing the need for energy and resource use in the first place. For example, designing cities so people don't need to drive as much, insulating homes so they consume less energy, or cutting back on unnecessary consumption. But going down this path disrupts consumption habits and social expectations, creating resistance from citizens and voters. Both strategies, therefore, require bigger institutional and cultural changes in how economies and societies are organized.

Ultimately, the central barrier to Paris implementation is not technological feasibility or economic viability, but the lack of political will. As UN Secretary-General António Guterres bluntly put it during a visit to Singapore in September, 2024: “*The only thing we need is the political will to use the instruments we already have to make sure that we reverse the present trends … that would lead to hell on Earth.”* This underscores that the barriers to effective climate action are not scientific, technological or financial, but political inertia, bolstered by entrenched vested interests. Political inertia is reinforced by vested interests and lobbying from fossil fuel, automotive, and defense industries, which continue to secure privileges and subsidies. This aligns with findings that climate misinformation and lobbying actively delay or weaken climate legislation, despite clear scientific and economic evidence supporting rapid decarbonization.

## Estimating priorities for mitigation

In a previous paper (25), by using a Global Calculator (GC) developed and tested by experts in several institutions, one of us has explored the relative contributions of different interventions for the mitigation of climate change. The reference trajectory was the International Energy Agency 4DS scenario (IEA4DS), which projects annual emissions of 53.6 GtCO_2_-eq in 2050. IEA4DS was selected, instead of more updated trajectories, because it approximates the current global trend in GHG emissions. Mitigation options in this framework refer to policy interventions implemented between 2015 and 2050, and their effect was expressed as a percentage reduction in all-sector emissions relative to IEA4DS 2050 levels (not relative to one sector only and to current emissions). The GC models these effects by simulating sectoral policies and actions, each described with four ambition levels, ranging from business-as-usual (level 1) to very ambitious but still feasible (level 4). Increasing all levers by +1 relative to the baseline results in a 91% reduction of emissions in 2050 compared with IEA4DS predictions for the same year, according to GC.

Within this framework, raising the ambition of the energy sector by +1 transition yields a reduction of 11.56 GtCO_2_-eq per year in 2050 compared to IEA4DS, or 21.6% of annual global emissions. This translates into substantial capacity increases by 2050 relative to IEA4DS: wind from 2,317 to 4,710 GW, hydroelectric from 1,750 to 2,101 GW, marine from 97 to 237 GW, solar remaining at **~**2,340 GW, geothermal from 172 GW to 289 GW, and electricity storage from 403 to 800 GW. An even higher ambition (+1.5 levels) would reduce 16.5 GtCO_2_-eq per year in 2050 (30.8% of all sector emissions).

Changes in land use and food systems can jointly deliver another 8–12 GtCO_2_e. By contrast, a large-scale shift to nuclear power contributes less. In the GC baseline (IEA4DS), nuclear capacity is 685 GW by 2050, rising to 1,030 GW under +1 ambition. This increase reduces emissions by only 3.5% in 2050, equivalent to about 1.9 GtCO_2_-eq, confirming the modest contribution of nuclear compared with renewables, diet, or land use. This is consistent with recent projections by the International Energy Agency (2025): in the Stated Policies Scenario (STEPS), nuclear capacity grows to ~650 GW by 2050, in line with the IEA4DS baseline; in the Announced Pledges Scenario (APS), capacity more than doubles by mid-century, supported by USD 120 billion annual investment by 2030; and in the Net Zero by 2050 Scenario, capacity exceeds 1,000 GW by 2050, similar to GC's +1 case. Even under these ambitious pathways, nuclear delivers only ~1–2 GtCO_2_-eq savings per year by 2050, reflecting long construction times and very high investment needs, including USD 670 billion cumulative for small and medium reactors by 2050. Comparably, the deployment of BECCS, which even under ambitious assumptions provides less than 3 GtCO_2_e annually by mid-century.

Mitigation in the transportation sector, as defined by the interventions proposed in the global calculator, is in a somewhat unique position, since it leads to modest reductions in emissions relative to the sector's total (around 3–5 GtCO_2_e by 2050, depending on technology deployment), but is associated with important health co-benefits. In the IEA4DS baseline, by 2050 the average passenger car with an internal combustion engine consumes 5.3 L/100 km, with only 2% of cars electric or hydrogen-powered. Under +1 ambition, this improves to 4.3 L/100 km and 10% electric/hydrogen cars. As a result, the transport lever reduces emissions by only −3.4% in 2050 (≈1–2 GtCO_2_-eq), while the related “travel” lever achieves a higher reduction of **–**12.7% (≈6.8 GtCO_2_-eq) due to modal shifts and behavioral changes. The conservative GC assumptions explain why transport appears modest in its contribution. By contrast, the International Energy Agency (2024) projects that in the STEPS scenario, 30%−35% of vehicles will be electric by 2050, and in the APS scenario, EVs reach 35% by 2035 and rise further by 2050, with hydrogen and synthetic fuels deployed for freight. This corresponds to ~2.1 GtCO_2_ savings in 2050 relative to STEPS, or ~3.9% of overall emissions under IEA4DS, still broadly consistent with the order of magnitude of the GC estimates.

The large relative contribution of diet to GHG mitigation reflects the land use change driven by demand for pasture and cropland to supply the animal feed for livestock production. The associated deforestation, as well as methane emissions from ruminant livestock, drive very substantial increases in GHG emissions. According to the global calculator, dietary shifts alone could deliver up to 6–7 GtCO_2_eq of avoided emissions per year by 2050, making it one of the most powerful single levers. Diet in the global calculator is characterized by human population dynamics and eating habits (calories consumed, quantity of meat, type of meat) while food reflects mainly agricultural production practices (crop yields, type of livestock feeding, treatment of wastes and residues). Both diet and food include impacts of transportation and other sources of energy use.

Similar calculations run by the non-profit organization Project Drawdown (26) confirm that the highest contributions to GHG reductions come from interventions in food, land use (including forest restoration and protection), and renewable electricity—especially solar and wind. In contrast, sectors like transportation, nuclear energy, and carbon capture technologies show lower mitigation potential, aligning with previous simulation-based findings. These results are in turn similar to estimates proposed by AR6 (5), and also in in a document by the UK BEIS (27).

These rough estimates give an idea of the type of modeling that is needed for a concerted, global and systematic committment to climate mitigation. While actions such as renewable energy deployment, electric mobility, or dietary shifts can be implemented nationally with substantial impact, others such as land use change, supply chains for critical minerals, and carbon removal technologies require international cooperation to avoid leakage effects, ensure equity, and optimize global benefits.

## Open questions for mitigation

When trying to assess priorities for mitigation based on quantitative estimates of sector-specific greenhouse gas emissions, one key limitation is that the evidence is often circumstantial, confidence intervals to assess variability of estimates are not available, and the evidence should be frequently updated. Building on the conceptual gaps outlined above, it is also crucial to recognize a number of unresolved technical issues that complicate the implementation and prioritization of mitigation strategies. The following is a non-exhaustive list of such open questions, which illustrate the complexity and interdependence of sectoral interventions.

- *Agricultural productivity*. Agriculture is responsible for 10.5% of global GHG emissions. In principle, industrial agriculture with extensive use of fertilizers and pesticides should be substituted by more sustainable agriculture, like organic or regenerative agriculture. However, there is no clear evidence that this can be done maintaining the same level of productivity per hectare of industrial agriculture, at least for some crops (e.g., grains). If productivity declines with organic/regenerative agriculture, this implies further extension of arable land, at the expense of biodiversity and carbon sinks (28).
- *Land-based carbon sinks*. In addition to degradation of forests and other non-agricultural areas (e.g., peatlands, wetlands) due to the demand for agricultural land, urbanization also limits land area that may serve as carbon sink. While it is clear that afforestation can play only a limited role in global mitigation efforts, systematic estimates of the carbon sink contribution of preserving forests vs. reforestation are limited (27).
- *Food for all*. The world population will still be increasing for many decades. This implies that food should become available for a larger population, which is at odds with a reduction in agricultural land and in the use of fertilizers, unless large increases in productivity per hectare are achieved. Systematic assessments of costs and benefits of precision agriculture are not available.
- *Electric cars*. Though GHG emissions are clearly reduced if internal combustion engines are substituted by electric engines (taking for granted a full decarbonization of electric energy production), technological improvements are needed on the capacity of batteries, disposal of waste, distribution of recharge facilities and on the reliance on rare elements, which may be associated to crucial geo-political problems. It is worth recalling, however, that *there is no free lunch*: internal combustion vehicles pose similar issues—resource extraction, pollution, and geopolitical dependency—but on a significantly larger scale. The problems associated with electric mobility are not unique, but rather represent a shift in the configuration and intensity of impacts.
- *Cement*. Cement is a source of hard-to-abate emissions. The world production and use of cement, due to rapid urbanization in many areas of the planet, has now reached a maximum and seems to start declining (29). An open question is whether cement can be produced in a more sustainable way, and even with negative emissions.
- *Nature-based solutions*. Nature-based solutions (NbS) are increasingly recognized as essential components of mitigation and adaptation strategies. However, several open issues remain. First, the permanence and measurability of carbon sequestration through NbS—such as afforestation, wetland restoration or soil carbon enhancement—pose scientific and methodological challenges, especially when used as offsets in carbon markets. Second, trade-offs may arise between maximizing carbon storage and preserving biodiversity or local livelihoods. Third, NbS are often promoted in isolation, while their integration with gray infrastructure and engineered solutions (e.g., for flood control or coastal protection) remains poorly implemented and evaluated. These complexities call for more systematic, context-specific assessments.
- *Carbon dioxide removal (CDR)*. While increasingly present in Net Zero scenarios, CDR options such as direct air capture, BECCS, or enhanced weathering raise major concerns about scalability, costs, energy use, land competition, and long-term permanence of stored carbon (30). The governance of their deployment, including who pays and who benefits, remains largely unresolved.
- *Green financing*. Finance is crucial to foster investments in the right sectors for an ecological transition. However, bonds are volatile and strongly influenced by political changes and market turmoils. Green bonds, that were becoming popular a few years ago, are now in decline because of political changes across the world, commercial wars, etc. More broadly, it is worth raising the question of how, and to what extent, private finance, including ESG investment frameworks and blended finance mechanisms, can be mobilized to support effective and equitable climate mitigation.

These issues not only reflect scientific or technological uncertainty, but also fuel ongoing public and policy debates. It is encouraging that the global conversation has largely shifted from questioning the existence or anthropogenic nature of climate change to discussing the types, intensity, and consequences of mitigation strategies. However, several of these topics, such as diet, carbon removal, electric vehicles, or the role of nuclear power, have become increasingly ideologically polarized, which risks further delaying the emergence of concerted, science-based solutions.

## Co-benefits, particularly for health, as an essential component of policies

Establishing priorities and solving open uncertainties are just the first steps to set a political agenda. Beyond establishing priorities and addressing uncertainties, it is essential to emphasize the political and social relevance of climate mitigation strategies. The following elements are crucial to building public consensus and legitimacy, and can also help unlock larger-scale financing by demonstrating broader co-benefits and alignment with development goals:

- Health co-benefits
- Co-benefits for natural systems (e.g., impact on biodiversity and ecosystem services such as air quality improvement, water regulation, etc.)
- Co-benefits in the economic, social (e.g., impact on inequalities), political, psychological domains
- Costs in the short and long-run, compared to a business-as-usual scenario, i.e., the cost of inaction or delayed mitigation (including choice of discount rates, costs of insurance …)
- Social acceptability of each measure (which requires using the tools of deliberative democracy).

Public health is a particularly important, but still underrated component in any assessment of the progress (or failure) of the Paris Agreement. While many technical and economic uncertainties remain, these are increasingly used as arguments to postpone or dilute climate action. However, mounting evidence shows that such delays come at a high cost to public health. Recent studies estimate that postponing mitigation efforts by just a few years could result in hundreds of thousands of additional premature deaths due to heatwaves, air pollution, and food insecurity worldwide (31, 32).

There were 80,000 excess deaths in Europe due to the heat wave in 2003 (33), then 63,000 in 2022 (34), while 400,000 Europeans have been affected by deadly floods and storms in 2024 (35). A recent study has suggested that 2,345,410 (95% confidence interval 327,603–4,775,853) climate change-related deaths will occur between 2015 and 2099 according to a business as usual scenario, only in Europe (based on 854 cities) (36). According to another study, in the absence of income-based adaptation, the global mortality rate attributable to temperature in 2080–2099 is expected to increase by 1.8% [95% CI 0.8%−2.8%] under a lower-emissions RCP 4.5 scenario and by 6.2% [95% CI 2.5%−10.0%] in the very high-emissions RCP 8.5 scenario relative to 2001–2020 (37).

These are still fragmentary estimates and an overall count for the world, that considers all health effects and not only heatwaves, is not available yet. The World economic forum has estimated that by 2050 there might be 14.5 million deaths in the world and $12.5 trillion in economic losses worldwide, attributable to climate change (38), but the figure looks rather speculative.

In our survey in (25) we aimed to identify examples of quantitative estimates of co-benefits associated with a multiplicity of mitigation efforts. In fact, the *Lancet* Pathfinder Commission has systematically analyzed the short-term health impacts of greenhouse gas mitigation (39). Pathfinder is based on specific mitigation actions that were either modeled or implemented in specific geographic areas and could be associated with health impacts; data were harmonized to increase comparability between studies. Their work incorporates an umbrella review of 57 original studies that were captured by 26 published systematic reviews. These studies assessed 196 mitigation actions in terms of both their health impacts and GHG emission effects. The mitigation actions described came mainly from high-income settings (129 actions, 65%), with a further 30 (15%) from upper-middle-income settings. Most of the evidence on health co-benefits was from the AFOLU sector with 103 out of 200 unique co-benefit estimates of mitigation actions, almost all of which focused on dietary changes; the next largest sector was transport with 43 actions (22%), followed by multisectoral interventions (i.e., interventions acting across multiple sectors). Pathfinder reported quantitative estimates in terms of Years of Life Lost per 100,000 population per year: air pollution was associated with 2,482 YLL/100,000/yr, Diet with 2,163, Physical activity with 164, and Injuries with 724. These estimates are valid for the mitigation actions and the countries for which evidence was available and are not necessarily generalizable.

Pathfinder is currently the most systematic evaluation of health co-benefits, but it also drew the attention to the limitations of the available literature, which is fragmented and very often local, i.e., not generalizable.

## Conclusions

Ten years after the Paris Agreement, while global targets remain essential as a compass, they are insufficient unless matched by credible pathways, sector-specific priorities, and a shared political vision. Uncertainty, whether scientific, technological, or geopolitical, must no longer serve as an alibi for inaction or delay. On the contrary, it should reinforce the case for immediate, precautionary policies that prioritize known co-benefits and reduce future risks.

Since Paris, technological progress has been remarkable. Today, renewable energy, electric mobility, and energy-efficient buildings are widely available, cost-competitive, and capable of decarbonizing approximately 60%−70% of global emissions. The remaining hard-to-abate sectors, including aviation (~3%) and energy-intense industries (~7%), are more difficult but not impossible: green hydrogen, synthetic fuels, and innovative materials are emerging solutions. In agriculture and land use, a 75% reduction in meat consumption could halve agricultural emissions. These advances confirm what the IPCC AR6 has stated clearly: “*we have solutions in all sectors to at least halve emissions by 2030*.” Financial resources are not the bottleneck either: “*while financial flows are a factor of three to six times lower than levels needed by 2030 to limit warming to below* 2 °C*, there is sufficient global capital and liquidity to close investment gaps. However, this relies on clear signaling from governments and the international community, including a stronger alignment of public sector finance and policy”* (40).

Hence, the barrier is political will, undermined by vested interests and lobbying power that slow adoption. Overcoming this impasse requires a multi-level strategy:

- Individual and grassroots collective action, often driven by health and wellbeing co-benefits, can shift social norms and create public demand for change.
- Private and corporate involvement, spurred by investment opportunities and economic levers, can accelerate innovation and the scaling of clean infrastructure.
- Top-down regulation and incentives, including fiscal reforms and clear rules, remain essential to align markets with climate objectives.

Public health is a particularly powerful, yet underused lever. Climate policies framed through health benefits mobilize public opinion, shape policy priorities, and foster cross-sectoral alliances. Positioning health at the core of climate action is not only ethically sound, but also politically and economically strategic.

Finally, the legal domain is emerging as a critical frontier. In July 2025, the International Court of Justice issued a landmark advisory opinion affirming that climate inaction may constitute an internationally wrongful act, exposing states to obligations of cessation, reparation, and protection of vulnerable populations. While non-binding, this ruling strengthens the alignment between law, science, and politics, opening pathways for accountability and justice in climate governance.

A successful climate strategy will therefore depend on our ability to integrate mitigation with equity, to link science with consensus-building, and to transform fragmented responses into coordinated, just transitions. This requires not only national commitment, but also stronger international governance capable of aligning financial flows, tracking progress transparently, and enforcing solidarity across countries and generations. The health of our planet, and of its people, demands nothing less.
